# Unveiling a *Listeria monocytogenes* Outbreak in a Rabbit Farm: Clinical Manifestation, Antimicrobial Resistance, Genomic Insights and Environmental Investigation

**DOI:** 10.3390/microorganisms12040785

**Published:** 2024-04-12

**Authors:** Inês C. Rodrigues, Marisa Ribeiro-Almeida, Leonor Silveira, Joana C. Prata, André Pinto de Carvalho, Carla Roque, João Paulo Gomes, Vítor Borges, Ângela Pista, Paulo Martins da Costa

**Affiliations:** 1 School of Medicine and Biomedical Sciences, University of Porto (ICBAS-UP), Rua de Jorge Viterbo Ferreira, 228, 4050-313 Porto, Portugal; icrodrigues@icbas.up.pt (I.C.R.); up201008465@edu.icbas.up.pt (M.R.-A.); joana.prata@iucs.cespu.pt (J.C.P.); andrepintodecarvalho@gmail.com (A.P.d.C.); 2 Interdisciplinary Centre of Marine and Environmental Research (CIIMAR), Terminal de Cruzeiros do Porto, de Leixões, Av. General Norton de Matos s/n, 4450-208 Matosinhos, Portugal; 3 UCIBIO—Applied Molecular Biosciences Unit, REQUIMTE, Laboratory of Microbiology, Department of Biological Sciences, Faculty of Pharmacy, University of Porto, 4099-002 Porto, Portugal; 4 National Reference Laboratory for Gastrointestinal Infections, Department of Infectious Diseases, National Institute of Health Doutor Ricardo Jorge, Avenida Padre Cruz, 1649-016 Lisbon, Portugal; leonor.silveira@insa.min-saude.pt (L.S.); carla.roque@insa.min-saude.pt (C.R.); angela.pista@insa.min-saude.pt (Â.P.); 5 i4HB—Institute for Health and Bioeconomy, University Institute of Health Sciences, Advanced Polytechnic and University Cooperative (CESPU), 4585-116 Gandra, Portugal; 6 UCIBIO—Applied Molecular Biosciences Unit, Translational Toxicology Research Laboratory, University Institute of Health Sciences (1H-TOXRUN, IUCS-CESPU), 4585-116 Gandra, Portugal; 7 NANTA Portugal, S.A., Rua da Estação, N° 157, 4630-221 Marco de Canaveses, Portugal; 8 Genomics and Bioinformatics Unit, Department of Infectious Diseases, National Institute of Health Doutor Ricardo Jorge, Avenida Padre Cruz, 1649-016 Lisbon, Portugal; j.paulo.gomes@insa.min-saude.pt (J.P.G.); vitor.borges@insa.min-saude.pt (V.B.); 9 Veterinary and Animal Research Centre (CECAV), Faculty of Veterinary Medicine, Lusófona University, 1749-024 Lisbon, Portugal

**Keywords:** *Listeria monocytogenes*, rabbit farm, antimicrobial resistance, clinical manifestation, genomic profiling

## Abstract

*Listeria monocytogenes* poses a threat to both human and animal health. This work describes an *L. monocytogenes* outbreak in a Portuguese rabbit farm, detailing the isolates’ clinical manifestations, necropsy findings, and phenotypic and genomic profiles. Clinical signs, exclusively observed in does, included lethargy and reproductive signs. *Post-mortem* examination of does revealed splenomegaly, hepatomegaly with a reticular pattern, pulmonary congestion, and haemorrhagic lesions in the uterus, with thickening of the uterine wall and purulent greyish exudates. Positive *L. monocytogenes* samples were identified in fattening and maternity units across different samples, encompassing does and environmental samples. Core-genome Multi Locus Sequence Typing (cgMLST) analysis confirmed the outbreak, with the 16 sequenced isolates (lineage II, CC31, and ST325) clustering within a ≤2 allelic difference (AD) threshold. Antimicrobial susceptibility testing for five antibiotics revealed that 15 out of 19 outbreak isolates were resistant to sulfamethoxazole-trimethoprim (SXT). Concordantly, all SXT-resistant sequenced isolates were found to exclusively harbour a plasmid containing a trimethoprim-resistance gene (*dfrD*), along with loci linked to resistance to lincosamides (lnuG), macrolides (mphB), and polyether ionophores (NarAB operon). All sequenced outbreak isolates carried the antibiotic resistance-related genes *tetM*, *fosX*, *lin*, *norB*, *lmrB*, *sul*, and *mprF*. The outbreak cluster comprises isolates from does and the environment, which underscores the ubiquitous presence of *L. monocytogenes* and emphasizes the importance of biosecurity measures. Despite limited data on listeriosis in rabbit farming, this outbreak reveals its significant impact on animal welfare and production.

## 1. Introduction

*Listeria* spp. are remarkably ubiquitous bacteria, able to survive and thrive in diverse conditions, including soil, water, and various food sources [1]. This genus comprises multiple species, among which *Listeria monocytogenes* (*L. monocytogenes*) stands out as a primary pathogenic strain with zoonotic implications [1]. This bacterial species has been detected worldwide in humans, animals, the environment, and food sources, emphasizing the importance of a holistic approach to health (One Health concept) [2]. Animal infection with *L. monocytogenes* is often related to dietary sources (stored forage) [3], while human infection typically results from the handling or consumption of uncooked or ready-to-eat foods [4]. This transmission is facilitated by the bacterium’s ability to grow and produce biofilm at refrigeration temperatures, thereby increasing the risk of cross-contamination during food processing and storage [5]. Even though disease occurrence is sporadic, an outbreak of listeriosis can originate severe clinical outcomes, such as septicaemia, meningitis, encephalitis, metritis, abortion, stillbirth, pyometra, and gastroenteritis in both humans and animals [6].

*L. monocytogenes* is actively monitored throughout the European food chain, from primary production to distribution, owing to its widespread presence as a foodborne pathogen [7]. According to the European Food Safety Authority (EFSA) Report from 2023, fish, fishery products, and products of meat origin (excluding sausages) exhibited the highest percentages of positive samples (2.6%, 2.5%, and 2.5%, respectively) [8]. However, in Portugal, previous studies have underscored an increased risk of *L. monocytogenes* in traditional cheeses (cured and fresh), possibly attributed to the Portuguese preference for this product at meals, posing a particular risk if they are made with raw milk or if not stored under the correct refrigeration conditions [9,10,11]. Despite a relatively low reported incidence rate in humans (0.62 per 100,000 population in 2022), there was an increase of 15.9% in the notification rate compared to the rate in 2021 [8]. Listeriosis causes the highest number of fatal cases (9.5%) of foodborne diseases in the EU, coupled with a notable rate of hospitalization (81.8%) [8].

In animals, listeriosis can impact various animals, including ruminants, birds, marine life, insects, and crustaceans [12]. Among these, sheep and goats presented the highest number of positive cases (2.5%), followed by cattle (1.2%) and pigs (0.35%) in 2022 [8]. Outbreaks of *L. monocytogenes* in rabbits have rarely been described, which might be attributed to undiagnosed or underreported cases, as listeriosis is not a notifiable infection [13]. Therefore, information on the prevalence of *L. monocytogenes* in rabbits worldwide is limited. Nonetheless, a prevalence of approximately 22% was reported in diseased rabbits in Egypt in 2016 [14]. Listeriosis may pose a threat to rabbit farms by having a negative impact on production since it could lead to the contamination of meat, representing a risk to human food safety [4,15,16,17]. It may cause additional economic losses attributed to illness and increased infertility and abortion rates, as observed in some animals [18]. Particularly in sheep and goats, the abortion rates may exceed 20%, and the incidence rate may reach 9% [19].

This work aims to describe an outbreak associated with *L. monocytogenes* infection on a rabbit farm in Portugal, including the clinical manifestation and diagnosis, antimicrobial susceptibility, and genomic analysis of *L. monocytogenes* isolates.

## 2. Materials and Methods

### 2.1. Rabbit Farm Description

A commercial rabbit farm located in Braga district, Portugal, houses 8000 fattening rabbits and 950 does. At 65 days of age, fattening rabbits reach an average weight of 17 to 19 kg per artificially inseminated doe. The does are artificially inseminated and undergo approximately 12–14 parturitions during their productive lifespan. They are also purchased from an external company for reproductive renewal every four or six months. The farm consists of two units, one for fattening and another for maternity, situated in the same building. Biosecurity measures include changing footwear, bathing, and changing clothing at the farm entrance and passing through a footbath. Each unit also has its footbath at the entrance. Sanitary voids are conducted every 42 days, aligning with the period when fattening rabbits are transported to the slaughterhouse. Afterwards, the unit undergoes sanitization, and the does are subsequently transferred to this facility. The farm operates with a core staff of two permanent employees, supplemented by short-time workers during peak demand. The rabbits are kept in suspended conventional cages and fed a commercial pelleted diet. Feeding for does is offered *ad libitum*, whereas fattening rabbits undergo controlled feed restriction. Water is provided *ad libitum* for all rabbits and sourced from a well that undergoes regular microbiological assessments. The rabbit farm is situated in a rural setting, where wild and domestic animals can be found. Particularly, a flock of six sheep often grazes the fields surrounding the rabbit farm. These animals have no direct contact with the rabbits.

### 2.2. Sample Collection

**Figure 1:**
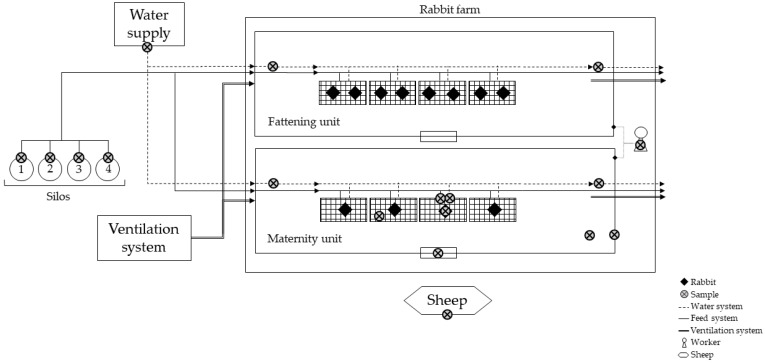
Schematic diagram of the rabbit farm and sample collection points.

The presence of *L. monocytogenes* was assessed in two matrices: animal and environment (Figure 1). The collected samples were refrigerated and promptly transported to the microbiology laboratory. Samples were processed within 2 h of collection.

#### 2.2.1. Does Samples

Freshly dead does (*n* = 10), exhibiting clinical signs such as anorexia, lethargy, vaginal purulent discharge, and intra-uterine foetal death, were examined in situ for *post-mortem* lesions. The management of the animals included in this study was conducted based on veterinary expertise, and necropsy procedures were performed during veterinary consultation, not being classified as animal experimentation following Directive 2010/63/EU on the protection of animals used for scientific purposes [20]. The uterus, liver, lungs, spleen, and stillborn kittens were collected and grouped in pools of 5 of each type in sterile containers for *Listeria* spp. isolation.

#### 2.2.2. Environmental Samples

Environmental samples were obtained from both fattening and maternity units. In the fattening unit, only drinking water was sampled, while in the maternity unit, sampling was also performed on surfaces. Particularly, five drinking water samples were collected in total, including well water and water from the initial and final dispenser lines in both units. A minimum of 1 L of drinking water was collected in a sterile plastic container. Surfaces from the maternity unit, such as feeders, drinkers, workers’ hands and footwear, walls, cages, window panels, and flooring, were also sampled. For each surface, a pool of 10 different swabs was created. In addition, feed from the four silos was collected using sterile plastic containers. Therefore, this study comprises 4 distinct environmental samples (Table 1).

#### 2.2.3. Nearest Livestock Population

The rabbit farm was surrounded by agricultural land, and the closest and most consistent livestock population nearby consisted of a herd of six asymptomatic sheep. One sheep from this herd was randomly selected for sampling, and both faeces and nasal secretions were collected for microbiological analysis (Table 1). A minimum of 100 g of fresh faeces obtained within 5 min were collected with sterile gloves and gathered in a sterile plastic container. Additionally, a pool of three nasal swabs was prepared and promptly refrigerated, along with the other collected samples (environmental and does samples).

### 2.3. L. monocytogenes Detection

Upon arrival, the 27 samples were immediately analysed for the presence of *L. monocytogenes*, according to ISO 11290 [21]. Briefly, samples were diluted 1:10 using Half-Fraser Broth (Biokar, Allone, France). Subsequently, all samples underwent homogenization in a Stomacher^®^ (Stomacher 400 circulator, Seward, West Sussex, UK) for 1 min. The pools of swabs (feeders, drinkers, workers’ hands and footwear, walls, cages, window panels, flooring, and sheep’s nasal secretion) were premoistened in the laboratory using 10 mL of buffered peptone water (Biokar). The pools were then vortexed and diluted 1:10 in Half-Fraser Broth and kept at 37 °C for 24 h. Compass Listeria Agar (Biokar) was then inoculated through the pour plate method with 1 mL from each suspension and was incubated at 37 °C for 24 h. Typical blue colonies were confirmed via a cross-streaking method using Palcam agar (Oxoid, Basingstoke, UK), incubated at 37 °C for 24 h. Up to five positive colonies per plate were stored in Tryptone Soya Broth (TSB, Biokar) with 20% glycerol in a −20 °C freezer. All frozen isolates were subsequently confirmed as *L. monocytogenes* through PCR targeting the hemolysin (*hly*) gene and subjected to antimicrobial susceptibility testing.

### 2.4. PCR Identification of L. monocytogenes

Prior to Polymerase Chain Reaction (PCR), the extraction of DNA was performed from fresh and pure colonies by suspending a colony in 1 mL of sterile ultrapure water and subsequent centrifugation for 2 min at 17,000× *g*. Afterwards, 200 μL of Instagene™ Matrix (Biorad, Hercules, CA, USA) was introduced into the suspension, followed by an incubation period of 30 min at 56 °C and 8 min at 100 °C, using a dry block heating thermostat (BIO TDB-100, Biosan, Riga, Latvia). The resulting supernatant was then stored at −20 °C. For PCR, the primers HlyA_R (5′-GCAACGTATCCT CCAGAGTGATCG) and HlyA_F (5′ GCAGTTGCAAGCGCTTGGAGTGAA) were used to ascertain the presence of the *hlyA* gene fragment (223 bp) [22,23]. The PCR reaction mixture, with a total volume of 25 μL, included 0.5 μL of Taq polymerase (DFS-Taq DNA Polymerase, Bioron, Römerberg, Germany), 2.5 μL of reaction buffer (10×, Bioron), 0.5 μL of dNTPs (10 μM, Bioron), 4 μL of each primer (10 μM), 8.5 μL of ultrapure water, and 5 μL of bacterial DNA. The PCR was performed in a thermal cycler (MyCycler™, Biorad, Hercules, CA, USA) at 95 °C for 3 min, followed by 30 cycles at 95 °C for 30 s, 58 °C for 30 s, and 72 °C for 1 min, and a final extension at 72 °C for 10 min. Subsequently, 5 μL of PCR products were subjected to electrophoresis in a 1.5% (*w*/*v*) agarose gel at 100 V for 45 min and stained with Green Safe Premium (NZYTech, Lisboa, Portugal). As a positive control, genomic DNA from *L. monocytogenes* CECT 911 (Spanish Type Culture Collection) was included.

### 2.5. Antimicrobial Resistance Testing

The resistance patterns of *L. monocytogenes* isolates identified via PCR were determined through the Kirby–Bauer method, following the European Committee on Antimicrobial Susceptibility Testing (EUCAST) guidelines [24]. Briefly, Mueller–Hinton agar (Biokar) supplemented with 5% (*v*/*v*) defribinated horse blood (Oxoid, Basingstoke, UK) and 20 mg/L β-NAD (Acros Organics, Geel, Belgium) was inoculated with a bacterial inoculum equivalent to 0.5 McFarland turbidity, followed by the placement of antibiotic discs. The plates were incubated at 35 °C with 5% CO_2_ for 18–20 h, and the diameter of the inhibition zones was measured in millimetres. The array of antimicrobial agents tested encompassed ampicillin (AMP, 2 µg), erythromycin (ERY, 15 µg), meropenem (MEM, 10 µg), penicillin (PEN, 1 unit), and sulfamethoxazole-trimethoprim (SXT, 1.25–23.75 µg), all sourced from Oxoid. The categorization of bacterial isolates into susceptible, intermediate, or resistant groups was based on EUCAST [25].

### 2.6. WGS Characterization

#### 2.6.1. Genomic DNA Extraction and Whole-Genome Sequencing (WGS)

Taking into account the sample type, location, and source, *L. monocytogenes* isolates from all matrices underwent WGS analysis. In the case of stillborn pools, only two out of the four strains were selected, as all isolates displayed identical antimicrobial phenotypes. The extraction of genomic DNA was carried out using the ISOLATE II Genomic DNA kit (Bio-line, London, UK) and quantified with the Qubit fluorometer (Invitrogen, Waltham, MA, USA) using the dsDNA HS Assay Kit (Thermo Fisher Scientific, Waltham, MA, USA), following the manufacturer’s guidelines. The NexteraXT library preparation protocol (Illumina, San Diego, CA, USA) was applied, and paired-end sequencing (2 × 150 bp) was performed on the NextSeq 2000 instrument (Illumina) following the manufacturer’s instructions.

#### 2.6.2. Bioinformatics Analysis

Read quality control, trimming, and *de novo* genome assembly were performed with the INNUca pipeline v4.2.2 (https://github.com/B-UMMI/INNUca, accessed on 10 November 2024) [26], using default parameters. In brief, FastQC v0.11.5 (http://www.bioinformatics.babraham.ac.uk/projects/fastqc/, accessed on 10 November 2023) and Trimmomatic v0.38 [27] were used for reads quality control and improvement, and de novo assembly was performed with SPAdes v3.14 [28]. Bowtie2 v2.2.9 [29] and Pilon v1.23 were applied for final assembly curation [30]. Kraken2 v2.0.7 [31] was used for the screening of species confirmation/contamination and mlst v2.18.1 (https://github.com/tseemann/mlst) (accessed on 29 November 2023) for Sequence Type (ST) determination. Core-genome Multi Locus Sequence Typing (cgMLST) was performed over the INNUca polished genome assemblies with chewBBACA v2.8.5 [32] using the 1748-loci Pasteur schema [33] available at the Chewie-NS website (https://chewbbaca.online/, downloaded on 23 June 2022) [34]. The cgMLST clustering analysis was performed with ReporTree v.2.0.3 (https://github.com/insapathogenomics/ReporTree, accessed on 15 November 2023) [35] using GrapeTree (MSTreeV2 method) [36], with clusters of closely related isolates being determined and characterized at distance thresholds of 1, 4, and 7 allelic differences (ADs). A threshold of seven ADs can provide a proxy for the identification of genetic clusters with potential epidemiological concordance (i.e., “outbreaks”) [37]. Reads and/or assemblies were screened for the presence of antimicrobial resistance genes with ABRicate v.1.0.1 (https://github.com/tseemann/ABRIcate) (accessed on 11 March 2024), though ReporType (https://github.com/insapathogenomics/ReporType) (accessed on 11 March 2024) [38] using NCBI AMRFinderPlus [39], CARD [40], Resfinder [41], and ARG-ANNOT [42] databases, and with the online BIGSdb-Lm (https://bigsdb.pasteur.fr/listeria/) (accessed on 11 March 2024) [43] and Centre for Genomic and Epidemiology (ResFinder 4.42, http://www.genomicepidemiology.org; accessed on 30 January 2024) tools.

Sequencing reads were deposited on the European Nucleotide Archive (ENA) under the bioproject PRJEB31216. Supplementary Materials Table S1 presents the accession numbers for each isolate.

## 3. Results

### 3.1. Case Description

**Figure 2:**
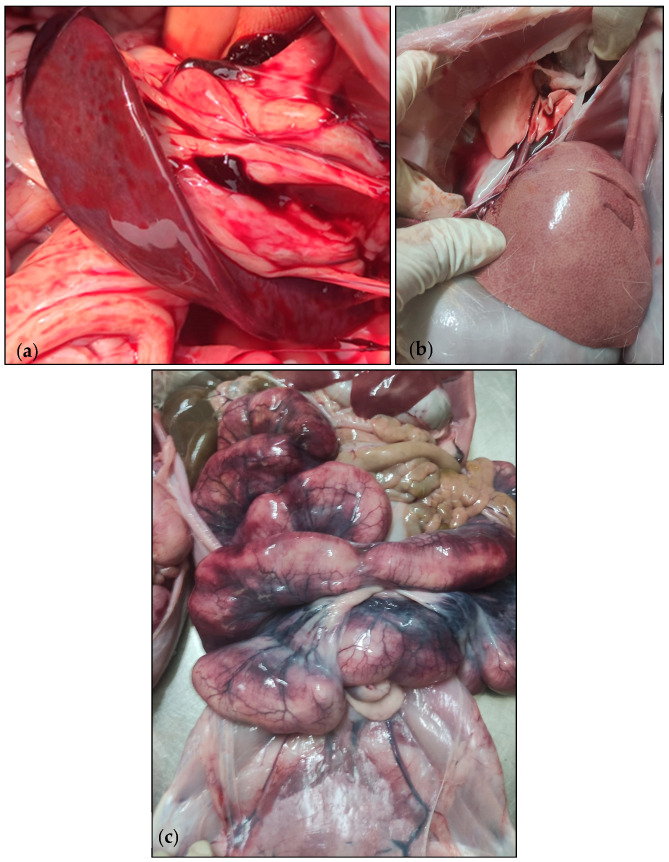
Necropsy lesions of rabbits. (**a**) splenomegaly, (**b**) hepatomegaly with a reticular pattern, and (**c**) haemorrhagic lesion in the uterus. Photographs kindly provided by the producer.

On 26 July 2023, an increased mortality rate among does was observed during the peripartum period, wherein 60 does died within 2 days, resulting in a 6% mortality rate. This is notably higher when compared to the historical records of this rabbit farm, which indicated an average peripartum mortality rate for does ranging from 2 to 3% over the past few years. In addition, 1200 stillborns were recorded. The clinical presentation predominantly consisted of anorexia, lethargy, vaginal purulent discharge, and intra-uterine foetal death. Eight days before the onset of these symptoms, does presented a respiratory infection and were receiving treatment with sulfadimethoxine-trimethoprim through their feed (4 kg/ton of feed). The antibiotic treatment lasted for 15 days and was still ongoing during the outbreak. It is worth noting that the fattening rabbits and the sheep did not display any clinical symptoms and were not subjected to antibiotic therapy. The *post-mortem* examination of the affected does reveal splenomegaly (Figure 2a), hepatomegaly with a reticular pattern (Figure 2b), and pulmonary congestion. Additionally, haemorrhagic lesions were observed in the uterus (Figure 2c), with thickening of the uterine wall and a mucosal covered in purulent greyish exudates. Nonetheless, the management of this clinical case was conducted on the veterinarian’s experience and routine therapy plan and was not influenced in any way by this study.

### 3.2. Detection of L. monocytogenes

*L. monocytogenes* was detected in 14 samples (14/27, 52%), including does and environmental samples (Table 2 and Figure S1). In the fattening unit, the water from the final dispenser line sample revealed the presence of *L. monocytogenes* but not the one collected in the initial dispenser line. In the maternity unit, samples from feeders, walls, cages, and all sampled organs from the does were also positive for *L. monocytogenes*. Additionally, the sheep faecal sample was positive for *L. monocytogenes*.

### 3.3. Phenotypic and Genotypic Characterization of L. monocytogenes Isolates

A total of 19 isolates were recovered from the 14 *L. monocytogenes* positive samples (Table 2). Among these, four isolates (4/19, 21%) exhibited susceptibility to all five tested antibiotics (AMP, ERY, MEM, PEN, and SXT), while fifteen isolates (15/19, 79%) showed resistance to sulfamethoxazole-trimethoprim (SXT). The only isolate obtained from the fattening unit also displayed resistance to SXT, while the isolate from the sheep’s faecal sample showed no antimicrobial resistance.

Genomic evaluation using WGS was performed on 16 out of the total 19 isolates, covering all matrices. Among the pools from the stillborn, only two isolates proceeded to WGS due to the similarity in susceptibility profiles across all isolates from these pools. Lineage II, CC31, and ST325 were identified among the 16 sequenced isolates (Table 3), with cgMLST confirming their clustering within a ≤2 allelic difference (AD) threshold, thus confirming the outbreak. In silico screening of antibiotic resistance genes (ARG) revealed the presence of several ARGs in all isolates, namely *tetM*, *fosX*, *lin*, *norB*, *lmrB*, *sul*, and *mprF* (Table 3). In addition, concordantly with the phenotypic observation, all sequenced isolates resistant to SXT (*n* = 12) were found to harbour a trimethoprim-resistance gene *dfrD*. Notably, this gene was detected in a plasmid exclusive of SXT-resistant isolates that also contained other loci linked to resistance to lincosamides (*lnuG*), macrolides (*mphB*), and polyether ionophores (NarAB operon). Intriguingly, the NarAB operon showed full identity to transferrable genes (accession MN590308) recently found in *Enterococcus faecium* [44], supporting horizontal gene transfer. Overall, phenotypic findings were supported by genomic results.

## 4. Discussion

*Listeria monocytogenes* is responsible for the highest number of fatal cases among foodborne diseases in Europe and contributes to a notable hospitalization rate in humans [8]. Particularly in Portugal, increased risks of *L. monocytogenes* have been identified in cheeses, posing a substantial risk to public health [9,11]. As a zoonotic pathogen, it significantly impacts various animal species, notably affecting sheep, goats, cattle, and pigs, which results in significant consequences for production, including economic losses due to illness, reduced fertility, and increased abortion rates [8,18]. Data about listeriosis in rabbits are limited, with only 14 articles retrieved from the Web of Science for the keywords “(listeria OR listeriosis) AND rabbit” (from 1988 to January 2024). The majority of the studies have focused on detecting *L. monocytogenes* in rabbit meat products at rabbit meat processing plants [15,17,45].

This study details the investigation of a listeriosis outbreak on a rabbit farm in Braga, Portugal. The does exhibited clinical signs during the peripartum period, including anorexia, lethargy, vaginal purulent discharge, and intra-uterine foetal death. A high mortality rate was also observed for both does and stillborn. Consequently, samples from does and the environment were analysed, and *L. monocytogenes* isolates underwent both phenotypic and genomic characterization. The clinical presentation observed in the does during the peripartum period was noteworthy. Sixty does died within a short span of two days, resulting in a mortality rate of 6%, which represents a significant reproductive impact and economic loss. This increase in mortality, associated with the recording of 1200 stillborns, raised concerns as historical records indicated an average peripartum mortality rate of 2 to 3% during this period. Interestingly, while does showed signs of illness, fattening rabbits had no clinical symptoms, which can be attributed to the pre-existing asymptomatic presence of *Listeria* spp. on the farm, as previously reported in other studies [46]. The combination of antibiotic therapy and the stress associated with the peripartum phase may have triggered the manifestation of *Listeria* spp. infection exclusively in does. Paralleled to pregnancy in humans, gravid animals face a heightened risk of contracting invasive listeriosis [47,48]. Also, the external introduction of this bacteria through the renewal of does from external companies cannot be excluded.

Notably, the rabbit farm has implemented biosecurity measures at three levels: access management, animal health management, and operation management [49]. These measures include strict protocols for personnel movement at the entrance and between areas of the rabbit farm, pest control measures, and microbiological controls of drinking water, theoretically making an outbreak of listeriosis highly improbable. Furthermore, the use of commercially processed pellets subjected to thermal processing (irradiation, pelleting, and extrusion) further reduces the likelihood of such an event occurring [50]. Although previous studies detected bacterial pathogens in animal feed, such as *Listeria* spp., *Escherichia coli*, *Clostridium perfringens*, and *Clostridium argentinense*, this study did not detect *L. monocytogenes* in feed samples [51,52].

*L. monocytogenes* isolates were found in both fattening and maternity units, encompassing does and environmental samples. Regarding the antimicrobial susceptibility profile, most *L. monocytogenes* isolates displayed resistance to sulfamethoxazole-trimethoprim (SXT) (79%), with this phenotype correlating with the presence of a *dfrD* gene carried by a plasmid that also contains *lnuG* and *mphB*. Although a plasmid carrying *dfrD*, *lnuG*, and *mphD* has been recently described in another CC31 isolate in France [53], we should highlight that, in our study, another resistance-related transferrable element was found to co-localize in the same contig, namely the *E. faecium* narasin-mediating NarAB operon [44]. It is likely that this multidrug resistance plasmid was differentially present in the bacterial population clones before the outbreak and that antibiotic selective pressure posed by SXT therapy during the outbreak led to the predominance of plasmid-bearing isolates in our sampling [54]. Of note, the sole isolate obtained from the water of the final dispenser line in the fattening units also exhibited resistance to SXT, which could be explained by posterior contamination of the drinking water shed by the workers, supporting cross-contamination between both units. This rapid and alarming spread of this bacteria raises biosecurity concerns.

Our findings also align with the frequent reports of trimethoprim and tetracycline resistance in *Listeria* spp. isolates from Europe and North America, encoded by the presence of *dfr* and *tet* genes, respectively [55]. All *L. monocytogenes* isolates obtained from the different samples analysed belong to lineage II, CC31, and ST325. For instance, while lineage II strains have historically been linked to sporadic clinical cases, there is a growing trend in certain countries, suggesting their emergence as a notable cause of clinical disease cases and outbreak events [56]. Nonetheless, previous studies have associated ST325 with low pathogenicity, as it was isolated from dairy processing plants in Lombardy but has not been identified in clinical sources [57,58]. The identification of a cluster on the rabbit farm indicates the bacteria’s circulation within the farm, possibly facilitated by workers’ movement, suggesting that the implemented biosecurity measures may require further enhancements. The detection of *L. monocytogenes* in various environmental samples, including walls, feeders, flooring, cages from the maternity unit, water from the fattening unit, and sheep faeces, highlights its ubiquitous presence and environmental persistence [59,60], creating uncertainty about its origin. Whether the environment serves as a potential source of *Listeria* spp. or if it is contaminated a posteriori remains unclear.

Despite the scarcity of data on listeriosis in rabbit farming, this outbreak highlighted the significant impact on animal welfare and production profitability. Moreover, considering the zoonotic potential of *Listeria monocytogenes*, continuous surveillance and monitoring are essential in rabbit farming production, as in other livestock sectors.

The rarity of such events highlights the need for further research to understand the factors contributing to listeriosis in rabbits and to implement effective preventive measures. The outbreak on this rabbit farm is a poignant reminder of the complex interactions between pathogens, host species, and environmental factors. The interplay between bacteria, transmission dynamics, and biosecurity measures requires further investigation to develop targeted strategies to mitigate the impact of listeriosis in rabbit farming.

## 5. Conclusions

The present study intended to investigate a listeriosis outbreak in a rabbit farm in Portugal, with clinical manifestations solely observed in does, displaying lethargy and reproductive signs. *Post-mortem* examination of does revealed structural alterations in the spleen, liver, lungs, and uterus. Our results identified *L. monocytogenes* in both fattening and maternity units, encompassing does and environmental samples. Through Core-genome Multi Locus Sequence Typing (cgMLST), the outbreak was confirmed, as all the 16 sequenced isolates (lineage II, CC31, and ST325) clustered within a ≤2 allelic difference (AD) threshold. Antimicrobial susceptibility testing further demonstrated that 15 out of 19 outbreak isolates displayed resistance to sulfamethoxazole-trimethoprim (SXT). This resistance was consistently associated with the presence of a plasmid carrying the trimethoprim-resistance gene (*dfrD*), along with loci linked to resistance against lincosamides (*lnuG*), macrolides (*mphB*), and polyether ionophores (NarAB operon). The identification of a cluster, encompassing isolates from both within and outside the farm, emphasizes the ubiquitous presence of *L. monocytogenes* and highlights the importance of biosecurity measures. This study highlights the importance of *L. monocytogenes* surveillance in rabbit farms, as it impacts animal welfare and animal production profitability.

## Figures and Tables

**Table 1:** Overview of the environmental samples analysed in this study.

Sample ID	Type of Sample	Sample Type
3572	Water	Well water
3573	Water	Water from the initial dispenser line
3574	Water	Water from the final dispenser line
3575	Water	Water from the initial dispenser line
3576	Water	Water from the final dispenser line
3577	Surface	Feeders
3578	Surface	Drinkers
3579	Surface	Hands of workers
3580	Surface	Walls
3581	Surface	Cages
3582	Surface	Window panels
3583	Surface	Footwear of workers
3584	Surface	Flooring
3585	Feed	Silo number 1
3586	Feed	Silo number 2
3587	Feed	Silo number 3
3588	Feed	Silo number 4
3597	Biological	Sheep’s faeces
3598	Biological	Sheep’s nasal swab

**Table 2:** Detection of *L. monocytogenes* using PCR.

Sample ID	Local	Source	Sample Type	Detection of *L. monocytogenes*
3572	Outside of the farm	Environmental	Well water	−
3585	Outside of the farm	Environmental	Silo number 1	−
3586	Outside of the farm	Environmental	Silo number 2	−
3587	Outside of the farm	Environmental	Silo number 3	−
3588	Outside of the farm	Environmental	Silo number 4	−
3597	Outside of the farm	Environmental	Sheep’s faeces	+
3598	Outside of the farm	Environmental	Sheep’s nasal swab	−
3579	Between units	Environmental	Hands of workers	−
3583	Between units	Environmental	Footwear of workers	−
3575	Fattening unit	Environmental	Water from the initial dispenser line	−
3576	Fattening unit	Environmental	Water from the final dispenser line	+
3573	Maternity unit	Environmental	Water from the initial dispenser line	−
3574	Maternity unit	Environmental	Water from the final dispenser line	−
3577	Maternity unit	Environmental	Feeders	+
3578	Maternity unit	Environmental	Drinkers	−
3580	Maternity unit	Environmental	Walls	+
3581	Maternity unit	Environmental	Cages	+
3582	Maternity unit	Environmental	Window panels	−
3584	Maternity unit	Environmental	Flooring	+
3589	Maternity unit	Animal	Pool of uterus	+
3590	Maternity unit	Animal	Pool of uterus	+
3591	Maternity unit	Animal	Pool of liver	+
3592	Maternity unit	Animal	Pool of lung	+
3593	Maternity unit	Animal	Pool of spleen	+
3594	Maternity unit	Animal	Pool of stillborns	+
3595	Maternity unit	Animal	Pool of stillborns	+
3596	Maternity unit	Animal	Pool of stillborns	+

+: present; −: absent.

**Table 3:** Overview of *L. monocytogenes* isolates’ phenotypic and genotypic characterization. An integrated view of results is described, listing the type of sample and antimicrobial resistance profile.

Strain ID	Local	Source	Sample	AST ^A^		ARG	Lineage	CC	ST
					Shared	Differentially Present			
3597F/2	Outside of the farm	Environmental	Sheep faeces	Susceptible	*tetM*, *fosX*, *lin*, *norB*, *lmrB*, *sul*, *mprF*	None	II	31	325
3576F/1	Fattening unit	Environmental	Water from the final dispenser line	SXT	*tetM*, *fosX*, *lin*, *norB*, *lmrB*, *sul*, *mprF*	*dfrD*, *mphB*, *lnuG*, NarAB operon	II	31	325
3577C/1	Maternity unit	Environmental	Feeders	SXT	*tetM*, *fosX*, *lin*, *norB*, *lmrB*, *sul*, *mprF*	*dfrD*, *mphB*, *lnuG*, NarAB operon	II	31	325
3580C/1	Maternity unit	Environmental	Walls	SXT	*tetM*, *fosX*, *lin*, *norB*, *lmrB*, *sul*, *mprF*	*dfrD*, *mphB*, *lnuG*, NarAB operon	II	31	325
3581C/3	Maternity unit	Environmental	Cages	Susceptible	*tetM*, *fosX*, *lin*, *norB*, *lmrB*, *sul*, *mprF*	None	II	31	325
3584F/2	Maternity unit	Environmental	Flooring	Susceptible	*tetM*, *fosX*, *lin*, *norB*, *lmrB*, *sul*, *mprF*	None	II	31	325
3589C/1	Maternity unit	Animal	Uterus	Susceptible	*tetM*, *fosX*, *lin*, *norB*, *lmrB*, *sul*, *mprF*	None	II	31	325
3590F/2	Maternity unit	Animal	Uterus	SXT	*tetM*, *fosX*, *lin*, *norB*, *lmrB*, *sul*, *mprF*	*dfrD*, *mphB*, *lnuG*, NarAB operon	II	31	325
3591C/1	Maternity unit	Animal	Liver	SXT	*tetM*, *fosX*, *lin*, *norB*, *lmrB*, *sul*, *mprF*	*dfrD*, *mphB*, *lnuG*, NarAB operon	II	31	325
3591C/2	Maternity unit	Animal	Liver	SXT	*tetM*, *fosX*, *lin*, *norB*, *lmrB*, *sul*, *mprF*	*dfrD*, *mphB*, *lnuG*, NarAB operon	II	31	325
3592C/1	Maternity unit	Animal	Lung	SXT	*tetM*, *fosX*, *lin*, *norB*, *lmrB*, *sul*, *mprF*	*dfrD*, *mphB*, *lnuG*, NarAB operon	II	31	325
3592C/2	Maternity unit	Animal	Lung	SXT	*tetM*, *fosX*, *lin*, *norB*, *lmrB*, *sul*, *mprF*	*dfrD*, *mphB*, *lnuG*, NarAB operon	II	31	325
3593C/1	Maternity unit	Animal	Spleen	SXT	*tetM*, *fosX*, *lin*, *norB*, *lmrB*, *sul*, *mprF*	*dfrD*, *mphB*, *lnuG*, NarAB operon	II	31	325
3593C/5	Maternity unit	Animal	Spleen	SXT	*tetM*, *fosX*, *lin*, *norB*, *lmrB*, *sul*, *mprF*	*dfrD*, *mphB*, *lnuG*, NarAB operon	II	31	325
3594C/1	Maternity unit	Animal	Stillborns	SXT	*tetM*, *fosX*, *lin*, *norB*, *lmrB*, *sul*, *mprF*	*dfrD*, *mphB*, *lnuG*, NarAB operon	II	31	325
3595C/1	Maternity unit	Animal	Stillborns	SXT	ND	ND	ND	ND	ND
3595C/2	Maternity unit	Animal	Stillborns	SXT	ND	ND	ND	ND	ND
3596C/1	Maternity unit	Animal	Stillborns	SXT	ND	ND	ND	ND	ND
3596C/2	Maternity unit	Animal	Stillborns	SXT	*tetM*, *fosX*, *lin*, *norB*, *lmrB*, *sul*, *mprF*	*dfrD*, *mphB*, *lnuG*, NarAB operon	II	31	325

AST, antimicrobial susceptibility testing; ARG, antimicrobial resistance genes; SXT, sulfamethoxazole-trimethoprim; ND, not determined; ST, sequence type; CC, clonal complex, ^A^, antibiotics tested: ampicillin, erythromycin, meropenem, penicillin, and sulfamethoxazole-trimethoprim.

## Data Availability

Sequencing reads were deposited on the European Nucleotide Archive (ENA) under the bioproject PRJEB31216. Supplementary Materials Table S1 presents the accession numbers for each isolate.

## Supplementary Materials

The following supporting information can be downloaded at https://www.mdpi.com/article/10.3390/microorganisms12040785/s1, Figure S1: Schematic diagram of positive samples in the rabbit farm, Table S1: Accession numbers of the genomic sequences for each *Listeria monocytogenes* strain.
