# Supplementary material for: Unveiling a Listeria monocytogenes Outbreak in a Rabbit Farm: Clinical Manifestation, Antimicrobial Resistance, Genomic Insights and Environmental Investigation
Source: Microorganisms. 2024 Apr 12;12(4):785. doi: 10.3390/microorganisms12040785 (PMC11051769; doi:10.3390/microorganisms12040785)
Supplement: Supplementary file 1 [file microorganisms-12-00785-s001.zip › microorganisms-2938937-supplementary.pdf]

Supplementary materials

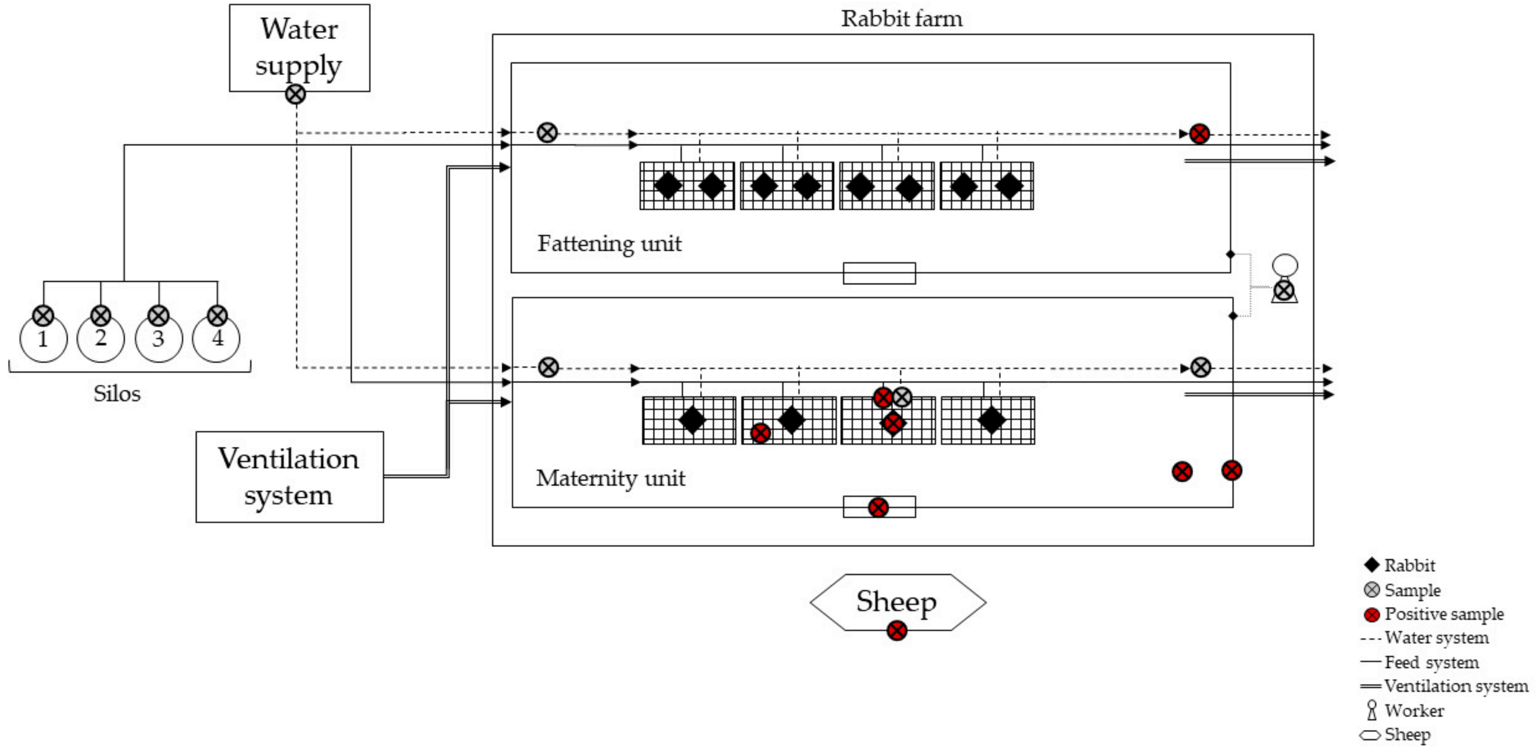

Figure S1 Schematic diagram of positive samples in the rabbit farm.

Table S1. Accession numbers of the genomic sequences for each *Listeria monocytogenes* strain.

SAMPLE	EnaProjectId	EnaSampleId	EnaExperimentId	EnaRunId
3594C/1	PRJEB31216	ERS18389171	ERX12096777	ERR12722210
3593C/5	PRJEB31216	ERS18389172	ERX12096778	ERR12722214
3597F/2	PRJEB31216	ERS18389173	ERX12096779	ERR12722219
3596C/2	PRJEB31216	ERS18389174	ERX12096780	ERR12722223
3593C/1	PRJEB31216	ERS18389175	ERX12096781	ERR12722215
3592C/2	PRJEB31216	ERS18389176	ERX12096782	ERR12722212
3592C/1	PRJEB31216	ERS18389177	ERX12096783	ERR12722207
3591C/2	PRJEB31216	ERS18389178	ERX12096784	ERR12722206
3591C/1	PRJEB31216	ERS18389179	ERX12096785	ERR12722217
3590F/2	PRJEB31216	ERS18389180	ERX12096786	ERR12722222
3581C/3	PRJEB31216	ERS18389181	ERX12096787	ERR12722209
3589C/1	PRJEB31216	ERS18389182	ERX12096788	ERR12722208
3584F/2	PRJEB31216	ERS18389183	ERX12096789	ERR12722216
3580C/1	PRJEB31216	ERS18389184	ERX12096790	ERR12722224
3577C/1	PRJEB31216	ERS18389185	ERX12096791	ERR12722221
3576F/1	PRJEB31216	ERS18389186	ERX12096792	ERR12722211
